# Precision therapeutics in non-scarring alopecia: a systemic genomic and pathway-based framework for targeted interventions

**DOI:** 10.5599/admet.3030

**Published:** 2025-12-16

**Authors:** Rinky Kapoor, Depti Bellani, Raji Patil, Debalina Bose, Madhuri Pola, Prashant Anilkumar Singh, Mamata Mishra, Debraj Shome

**Affiliations:** 1 Department of Dermatology, Cosmetic Dermatology & Dermato-Surgery, The Esthetic Clinics, Mumbai, Maharashtra, India; 2 Department of Medical Affairs, Esthetic Creations International Pvt. Ltd. (ECIPL), Mumbai, Maharashtra, India; 3 Department of Research, The Esthetic Clinics Clinical Research Organization (TECCRO), Mumbai, Maharashtra, India; 4 Department of Facial Plastic Surgery & Facial Cosmetic Surgery, The Esthetic Clinics, Mumbai, Maharashtra, India

**Keywords:** Androgenetic alopecia, alopecia areata, genome-wide association studies and next-generation sequencing, wingless-related integration sit/beta-catenin signalling pathway, Janus kinase-signal transducer and activator of transcription pathway, artificial intelligence

## Abstract

**Background and purpose:**

Non-scarring alopecia, principally androgenetic alopecia and alopecia areata is highly prevalent and psychologically burdensome; androgenetic alopecia is androgen-driven, whereas alopecia areata is autoimmune. This review synthesizes genetic architecture and pathway biology to outline a precision framework for targeted interventions.

**Experimental approach:**

We reviewed full-text studies from the past decade across PubMed, Web of Science and Google Scholar, applying explicit inclusion/exclusion criteria; emphasis was placed on Genome wide association studies and Next generation sequencing findings, immune and androgen-axis biology, environmental modifiers, and therapeutic evidence (conventional, targeted, and regenerative), alongside artificial Intelligence-enabled diagnostics.

**Key results:**

Androgenetic alopecia risk converges on androgen-receptor signalling and related loci, with perifollicular inflammation and oxidative stress as modifiers; finasteride remains a cornerstone therapy. Alopecia areata reflects polygenic immune dysregulation (*e.g.* Human leukocyte antigen/cytokine axes) with Janus Kinase-pathway inhibition yielding robust regrowth; across phenotypes, wingless-related integration sit/β-catenin and stem-cell programs are central targets. Regenerative options (Protein Rich Plasma, stem-cell/exosome approaches) and artificial Intelligence-assisted stratification are emerging adjuncts.

**Conclusion:**

A pathway-guided, genotype and phenotype-informed strategy, targeting the androgen axis for androgenetic alopecia, immune circuits for alopecia areata, and adding regenerative or microenvironmental therapies where indicated-promises earlier diagnosis and more durable, individualized outcomes, especially as genome-wide association study/next-generation sequencing and artificial Intelligence tools are integrated into care.

## Introduction

Alopecia is a genetic disorder, often referred to as a hair loss condition, which affects 2.1 % of people across the world [1]. Based on its nature of reversibility, alopecia is further classified into scarring and non-scarring alopecia. Scarring alopecia results in the permanent loss of hair because of irreversible damage to the hair follicles, while in non-scarring alopecia, the hair follicles remain intact, and proper treatment based on the cause results in hair re-growth [2]. Of the various kinds of non-scarring alopecia, the most popular non-scarring alopecia are androgenetic alopecia (AGA) and alopecia areata (AA) [3].

Male- or female-pattern baldness is the most prominent characteristic of AGA, driven by androgenic effects at the genetic level, in which the anagen phase is shortened, leading to follicular shrinkage [4]. It was reported from various studies that nearly 74.8 % of men and 40 % of women will experience this condition in their lifetimes. Unlike AGA, which is caused by androgens, alopecia areata is an autoimmune condition in which the immune system attacks the hair follicles, causing patches [5]. The exact cause of AA remains unclear; however, environmental factors, along with weakened immunity, contribute to hair loss. AA is most commonly observed in youngsters and has a significant impact on their physical appearance, a major factor accounting for both emotional distress and psychological problems [6].

In-depth research into the genetic factors underlying non-scarring alopecia will help tailor targeted therapies for hair regrowth. The literature indicates that AA is caused by deregulation of human leukocyte antigen (HLA) genes, changes in cytokine production, or T-cell activation, leading to follicular damage [7]. Researchers are focusing on targeting the Janus Kinase pathways as a tailored solution to treat individuals with AA [8]. This will be one of the safest and most promising approaches to using corticosteroids to promote hair regrowth. Genetic variations in androgen receptors have led to AGA. These biomarkers can be used to diagnose and treat AGA. Among the available medications, finasteride is the most commonly prescribed for AGA [9].

Even though various genetic factors contribute to hair loss, it is attributed to the combined effects of medications, nutrition, and environmental factors. Some molecular signalling pathways that can be targeted for hair growth include Wnt signalling pathway factors, hair follicle stem cell regulation pathways, etc. [10]. Technological advances, such as genetic screening, the integration of Artificial Intelligence (AI), and precision medicine, will enable individualized treatment for the management of non-scarring alopecia [11]. The present study explores in detail the understanding mechanisms of various genetic markers, associated pathways, psychological impact, and tailored therapies for curing non-scarring androgenic alopecia.

## Genetic basis for the non-scarring alopecia

### Search strategy

Articles related to non-scarring alopecia, genetic and molecular pathways, and associated treatment papers are retrieved from databases such as PubMed, Web of Science, and Google Scholar. The keywords used for the identification of the relevant papers are “alopecia”, “hair loss”, “hair regrowth”, “tailored treatments”, “technology”, “traditional methods”, “Genetic Markers”, “molecular pathway signalling” and “herbal therapies”. To understand molecular data analysis, some studies on microarrays and gene expression profiling in non-scarring alopecia were also utilised.

### Inclusion and exclusion criteria

Studies on alopecia, specifically non-scarring alopecia, are considered. There are a few studies related to randomised controlled trials, case studies, and quantitative studies. The articles that are full-length and freely available in the above database were chosen for this study. Articles from the past 10 years were collected, and further analysis was conducted. Studies older than 10 years were excluded. Studies of hair loss without significant details on non-scarring alopecia were excluded. Studies on hair regrowth supplements are also excluded. In the past, genetic analysis has enabled detailed study of disease pathophysiology and helped tailor personalised treatments. As the literature indicates that AA and AGA are inherited hair disorders, genetic analysis will help prevent hair loss.

**Figure 1.**
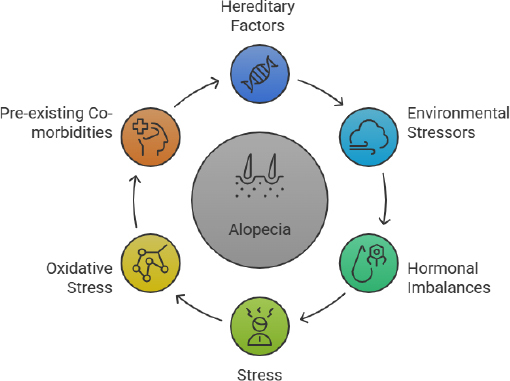
Causes of alopecia

Technological advancements have provided evidence of the genetic variants and their loci that influence the type of alopecia [12]. Single-gene-related alopecia is known as monogenic, whereas multiple-gene-related alopecia is known as polygenic [13]. Alopecia is a complex hair disorder that can be triggered by environmental factors in addition to genetic predisposition [14]. This, in turn, will affect hair follicle cycling, the autoimmune response, and hormonal dysregulation, thereby impacting individuals' psychological health. Some of the crucial techniques that have become popular in recent times include genome wide association studies (GWAS) and next-generation sequencing (NGS) to uncover the causes of different types of non-scarring alopecia [15]. Various researchers are working on different aspects related to immune changes, environmental impact, pre-existing disorders, genetic analysis (Figure 1), and technology integration to identify potential targets for diagnosing and treating non-scarring alopecia.

## Environmental and psychological factors in alopecia areata

**Figure 2.**
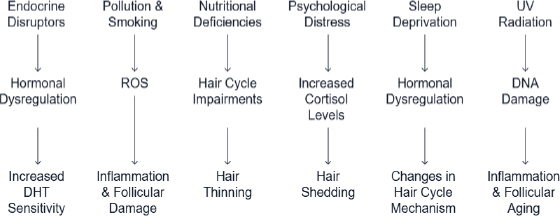
Impact of environmental stressors on AGA

The most common environmental factors that trigger AGA, in addition to genetic changes, include hormonal changes, pollution, oxidative stress, nutritional deficiencies, sleep disturbances, psychological distress, smoking, alcohol, and exposure to UV radiation (Figure 2) [16]. Endocrine-disrupting chemicals, such as phthalates and pesticides, mimic androgenic pathways, increasing dihydrotestosterone (DHT) sensitivity, thereby causing follicular miniaturization, weak, thin hair, and patterned baldness [17]. One of the most commonly recommended AGA treatments is finasteride or dutasteride [18]. Smoking and air pollutants generate reactive oxygen species (ROS) in the hair follicles and account for their damage. High-fat, high-sugar, and low-protein and mineral foods increase androgen production, impair follicular function, and induce follicular inflammation, respectively. Psychological stress increases cortisol levels, leading to androgen sensitivity and inflammation. Disturbances in the sleep cycle affect the follicular cells' circadian regulation. Long-term exposure to UV causes scalp inflammation, follicular stem cells damage and can alter the patterns of aging [19].

Alotiby *et al.* [20] presented a case study of a 28-year-old female diagnosed with AA, with no history of autoimmune disorders, whose condition was exacerbated by emotional stress.

Their research focused on the role of psychological distress as an environmental factor triggering AA. The researchers explored the combination of minoxidil treatment and stress reduction techniques, which resulted in improved hair growth among individuals suffering from psychological distress. This suggests that addressing emotional well-being alongside traditional therapies can improve outcomes for patients with AA. Increased stress activates the hypothalamic-pituitary-adrenal (HPA) axis, causing hair follicle damage. Oxidative stress is the body’s inability to neutralise the reactive oxygen species, which in turn accounts for the hair follicle death and inflammation.

## Immune mechanisms and inflammatory pathways in androgenetic alopecia and alopecia areata

**Figure 3.**
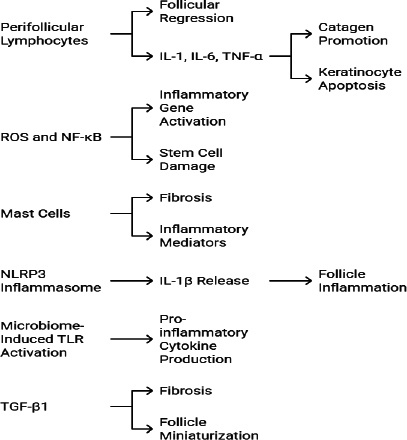
Immune pathways involved in AGA

AGA is primarily caused by the genetic predisposition and androgen metabolism. Recent research has revealed that immune and inflammatory pathways are also involved in the progression of AGA (Figure 3). Perifollicular inflammation is the most commonly observed finding in biopsies of AGA patients. The upregulation of pro-inflammatory cytokines such as IL-1α, IL-6, and TNF-α increases baldness and catagen-promoting effects. Increased ROS activates the transcription factor NF-κB, which, in turn, causes inflammation.

The studies of Cuevas-Diaz Duran *et al.* [21] have provided evidence that infiltration of T cells and Mast cells in the follicular areas promotes fibrosis. Zong *et al.* [22] reported that NLRP3 inflammasome activation, associated with the innate immunity, causes AGA. This process involves the activation of caspase-1, which, in turn, triggers inflammatory pathways. Some microbial infections, particularly those involving *Cutibacterium* spp. and *Malassezia* spp., stimulate TLR2 and TLR4, thereby activating inflammatory pathways and inducing the release of inflammatory cytokines that contribute to AGA [23].

Lina Alhanshali *et al.* [1] provided a comprehensive review of the immune mechanisms underlying AA, particularly the downregulation of major histocompatibility complex-I (MHC), which plays a key role in immune responses. Healthy hair follicles secrete immune-privileged factors, such as IL-10, αMSH, and TGF-β1, to protect against autoimmune attacks; however, this process is impaired in AA. Elevated interleukin (IL-2, IL-4, IL-15) levels have been observed in AA patients, with a subsequent decrease after treatment [1]. Additionally, increased expression of Toll-like receptors (TLR7 and TLR9) has been observed in AA patients, suggesting that targeting these receptors could mitigate disease severity. Further research into the genetic and molecular mechanisms of AA has also focused on the role of autophagy in disease development. Yi Lin *et al.* [24] demonstrated that disruption of the autophagy mechanism and accumulation of SQSTM1 proteins contribute to AA, and pharmacological induction of autophagy could potentially prevent scarring in affected individuals. The pathways involved in non-scarring alopecia were tabulated in Table 1.

**Table 1.** Pathogenic pathways and recommendation for non-scarring alopecia

Pathway/factor Involved	Recommendation	Ref.
Immune factors forkhead box P3 (FOXP3), inducible T-cell costimulator ligand (ICOSLG), major Histocompatibility complex class I-related sequence A (MICA), Interleukin 7 receptor alpha (IL7RA), HLA subtypes, *etc*	Immune dysregulation causing AA, targeting the inhibitors for these genes will prevent AA	[12]
Genetic variations in protein tyrosine phosphatase non-receptor type 22 (PTPN22), HLA-DRB1	JAK inhibitors	[12]
Interleukin-13 (IL-13), C-type lectin domain family 16 member A (CLEC16A) susceptibility loci, cytotoxic T-lymphocyte-associated protein 4 (CTLA4) pathways	Exploring CTLA4 and T cell populations for AA treatment strategies	[13]
Environmental factor- psychological stress	Stress management + medications	[20]
Arginine deficiency- dysregulation of mechanistic target of rapamycin (mTOR) pathway,	Clinical intervention for mTOR pathway alterations will prevent AA	[25]
Interleukin-33 (IL-33) as a link between asthma and AA	IL-33 inhibitors	[26]
Metabolic profiling	Need to study further to understand interactive effects	[26,27]
Impact of gut microbiome and metabolites	In-depth understanding of the gut microbiome is recommended	[28]
Immune dysregulation cluster of differentiation 24 (CD24), C-C chemokine receptor type 2 (CCR2) transcriptional "hot spots"	Genetic profiling + transcriptional analysis for early diagnosis and prevention of AA	[29]
Dysregulated hair follicle stem cells (HFSCs) + environmental factors	Targeting HFSCs will cure non-scarring alopecia	[30]
Decreased leukocyte telomere length (LTL)	Inhibitors against LTL prevention will prevent AA	[31]
Immune dysregulation-CD8A, phosphatidylinositol-4,5-bisphosphate 3-kinase catalytic subunit gamma (PIK3CG), src kinase associated phosphoprotein 1 (SKAP1)	Cluster of Differentiation 8 alpha chain (CD8A) and immune checkpoint pathway inhibitors	[32]
Immune collapse-plasmacytoid dendritic cells (pDCs), IFN-γ	Clinical intervention to pDCs will prevent AA	[33]
HLA associations and cytokines (IL-2, IFN-γ, IL-10, *etc*.)	JAK inhibitors can be used to treat AA	[34]
Metabolic profiling	Metabolites can be used as biomarkers for AA	[35]
Immune cell accumulation, neuroendocrine system disruption, stress hormones	Neurocrine mediators	[36]
Oxidative stress leading to DNA damage and antioxidants	Vitamin supplementation recommendations	[37]
Dysregulated autophagy	Autophagy prevention will prevent AA	[38]
Vitamin D deficiency and zinc levels	Supplements for vitamin D	[39]

## Genetic and molecular insights

Genetic research has further advanced the understanding of AGA and AA. Y. Li *et al.* [25] explored the role of impaired arginine metabolism in androgenetic alopecia (AGA), finding that an arginine deficiency and increased levels of ornithine disrupted the mTOR pathway, a key regulator of hair follicle development. By supplementing arginine and using dietary supplements, hair follicle regrowth was promoted in *ex vivo* experiments, suggesting that targeting metabolic pathways could be a therapeutic strategy for AGA [40].

In a broader genetic context, Mendelian randomization (MR) studies have provided insights into the genetic risk factors of AA. Few researchers have performed MR and genome-wide association studies (GWAS) to investigate the relationship between asthma and AA, identifying interleukin IL-33 as a mediator [41]. Their findings indicate that individuals with asthma have an increased risk of developing AA, thus highlighting the interconnectedness of immune-related diseases. Additionally, Yimei Du *et al.* [42] used a two-sample MR approach to examine the impact of approximately 452 metabolites on AGA. Their analysis identified heme and 2-palmitoyl-glycerophosphocholine as risk factors for AGA, while scyllo-inositol and alpha-ketoglutarate were found to have protective effects. This study underscores the importance of metabolites in the progression of AGA, further suggesting potential biomarkers for diagnosis and treatment.

Androgenetic alopecia (AGA) is a multifactorial condition characterized by progressive hair follicle miniaturization in genetically predisposed individuals under the influence of androgens. While the role of dihydrotestosterone (DHT) is central to its pathophysiology, mounting evidence underscores a complex genetic architecture involving several key genes and signalling pathways that regulate follicular response to androgens.

The androgen receptor (AR) gene, located on the X chromosome (Xq11-12), has emerged as a major genetic determinant of AGA. Hibberts *et al.* [30] demonstrated significantly higher levels of AR expression in dermal papilla cells from balding scalp compared to non-balding regions, establishing a functional link between AR density and follicular sensitivity to androgens. Subsequent genetic studies identified several single-nucleotide polymorphisms (SNPs) and triplet-repeat polymorphisms in the AR gene associated with early-onset AGA. However, not all polymorphisms exert a pathogenic effect; for instance, Ellis *et al.* [43] reported that the AR polyglycine repeat variant does not confer increased susceptibility. Beyond AR, the EDA2R gene, also located on the X chromosome, has been implicated in AGA. Prodi *et al.* [44] identified a strong association between EDA2R polymorphisms and male pattern baldness, potentially through linkage disequilibrium with AR, further highlighting the importance of maternal inheritance patterns in AGA.

Genome-wide association studies (GWAS) have broadened our understanding of AGA by revealing additional risk loci. Heilmann *et al.* [45] identified four significant loci at 2q35, 3q25.1, 5q33.3 and 12p12.1, implicating genes such as WNT10A, which plays a role in hair follicle cycling and differentiation. Similarly, Shimomura *et al.* [46] linked APCDD1, a known Wnt signalling inhibitor located at 18p11.2, to hereditary hair loss. These findings support the crucial role of Wnt/β-catenin signalling in maintaining follicular integrity, a hypothesis reinforced by Leirós *et al.* [47], who demonstrated androgen-mediated suppression of Wnt signalling in dermal papilla cells.

Additional gene expression profiling studies provide insights into regional differences in scalp gene activity. Mirmirani *et al.* [48] identified 38 differentially expressed genes between the vertex and frontal scalp in AGA patients, while Midorikawa *et al.* [49] reported a distinct expression profile in dermal papilla cells from balding regions. These findings suggest localized genetic regulation in response to androgen exposure. The role of prostaglandin metabolism has also garnered interest. Garza *et al.* [50] found elevated levels of prostaglandin D2 (PGD2) and its synthesizing enzyme PGDS in balding scalp regions, implicating PGD2 as an inhibitor of hair growth via premature catagen induction. However, Heilmann *et al.* [51] argued against a genetic basis for PGD2’s involvement due to the lack of GWAS association near PGDS loci.

Finally, genetic variations in steroid 5 alpha-reductase 1 (SRD5A1) and steroid 5-alpha-reductase type 2 (SRD5A2), encoding the two isoforms of 5α-reductase, contribute to increased DHT formation in balding areas, amplifying androgenic effects at the follicular level [52,53]. Together, these findings demonstrate that AGA arises from a complex interplay among androgenic stimulation, genetic predisposition, and modulation of signalling pathways. Future studies integrating GWAS, epigenetic profiling, and functional genomics will be instrumental in unravelling the precise molecular etiology and identifying novel therapeutic targets.

## Pathways and mechanisms of androgenetic alopecia and alopecia areata

AGA is a multifactorial disorder that is driven by genetic as well as various environmental stressors. Kwack *et al.* [54] have described that the DHT binding to the dermal papilla increases the levels of TGF-β1 and DKK-1, in turn suppressing the Wnt/β-catenin signalling pathway, preventing the follicular regeneration. The scalp affected by AGA will exhibit perifollicular inflammation due to elevated levels of pro-inflammatory cytokines, leading to immune-mediated damage to hair follicles. Oxidative stress causes stem cell dysfunction and activates the NF-κB signalling pathway [55].

### Dihydrotestosterone and Wingless-related integration site suppression cycle androgenetic alopecia

**Figure 4.**
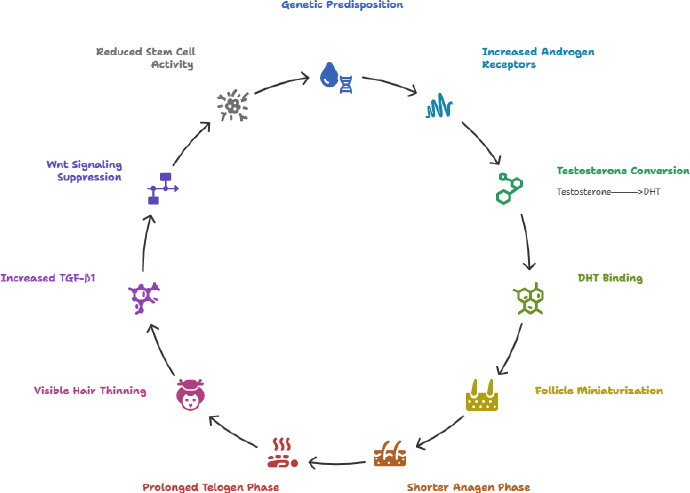
DHT-Wnt suppression cycle - AGA

DHT, an androgen hormone, is one of the primary culprits of AGA in individuals with a hereditary predisposition to the condition. Those with AGA heredity tend to have more androgen receptors in hair follicles, which are directly associated with increased androgenic activity. Within the hair follicle, the enzyme 5α-reductase catalyses the conversion of testosterone to DHT. The DHT formed then binds to androgen receptors, initiating an intracellular cascade that leads to hair follicle miniaturization, a shortened anagen phase, and a prolonged telogen phase, ultimately resulting in visible hair loss. This process is further exacerbated by the production of transforming growth factor-beta 1 (TGF-β1), triggered by DHT, which promotes hair follicle regression and, in turn, reduces follicular activity, thereby contributing to progressive hair loss (Figure 4).

In rare chronic inflammatory conditions, permanent hair follicle damage is observed due to fibrosis. There is a need to develop targeted therapies that not only address androgen activity but also immune/inflammatory responses. Various molecular pathways have been implicated in the pathogenesis of AA. Zihyu Liu and Xiaoyan Liu [56] reviewed several critical signalling pathways involved in AA, including JAK-STAT, TGF-β, and Wnt/β-catenin pathways.

These pathways are dysregulated in AA, leading to impaired hair follicle growth and immune responses. Additionally, factors such as oxidative stress, gut microbiome imbalances, and intestinal barrier disruption have been identified as contributors to disease progression. The authors proposed that a combination of targeted treatments addressing these pathways could improve outcomes for AA patients.

Moreover, Baoyi Liu *et al.* [57] conducted a microarray analysis to identify prognostic signatures associated with scalp diseases like AA. Their findings indicated upregulation of T-cell chemotaxis and interferon-β responses, along with downregulation of keratin filaments, suggesting that these changes may serve as biomarkers of AA. Coda & Sinha [58] further explored the clinical heterogeneity of AA, identifying dysregulated genes and transcriptional "hot spots" by integrating genome-wide transcriptional and genetic data. This highlights the importance of combining genetic and transcriptional analyses to identify novel biomarkers and improve clinical interventions. The genes that modulate AGA and AA are shown in Table 2.

**Table 2.** Genes responsible and associated pathways for modulating androgenetic alopecia

AGA/AA	Gene	Primary pathway getting altered	Detailed pathway analysis
AGA	AR (androgen receptor)	Androgen signalling [28-31,37,45,59]	Shorter CAG repeats + rs6152 SNP → ↑AR activity & DHT binding → ↑Androgen-responsive gene expression → Follicle miniaturization → Hair loss-AGA
	SRD5A2-5α-reductase type 2	Androgen signalling [28,46,60]	V89L (rs523349) ± A49T → ↑5α-reductase activity → ↑DHT in follicles → Miniaturization → Hair loss-AGA
	Dickkopf-related protein 1 (DKK1)	Wnt/β-catenin Signalling [39,54]	↑DKK1 → Binds LRP5/6 → Blocks Wnt signalling → ↓β-catenin activity → Impaired follicle regeneration → Hair loss-AGA
	SFRP1/2	Wnt/β-catenin Signalling [30,47]	↑SFRPs → Bind Wnt ligands (decoy receptors) → Inhibit Wnt signalling → ↓Follicle activation → Hair loss-AGA
	WNT10A	Wnt/β-catenin Signalling [31,32,34,61,62]	WNT10A →↑Wnt signalling→stabalises β-catenin→ ↑Follicle activation→ Prevents hair loss-AGA If mutation happens: rs7349332 (SNP mutation)-WNT10A variants →↓Wnt signalling→↓Follicle activation→Promotes hair loss-AGA
	MTOR, SLC7A1, ARG1/ARG2	mTOR/Arginine Metabolism [63]	SNPs → ↓Arginine uptake + ↑Ornithine → ↓mTOR signalling → ↓Follicle cell proliferation → Hair loss-AGA
	NFKB1, RELA, TNF, IL6, SOD2, *etc.*	NF-κB signalling (Inflammation/stress) [42]	Oxidative stress → NF-κB activation → ↑Pro-inflammatory genes (*e.g.* IL6, TNFα) → Stem cell dysfunction & follicle damage → Hair loss-AGA
	PTGDS (prostaglandin D2 synthase)	Prostaglandin pathway [50]	↑PTGDS → ↑PGD2 in follicles → Binds GPR44 receptor → Inhibitory signalling → Hair growth suppression → Hair loss-AGA
	PTGDR2 (GPR44)	Prostaglandin pathway [50]	↑PTGDR2 expression → ↑Sensitivity to PGD2 → Inhibitory signalling → Early catagen/telogen → Hair loss-AGA
	SRD5A1	Androgen signalling [64]	Converts testosterone to DHT; ↑SRD5A1 expression in balding follicles → ↑DHT production → Enhanced AR activation → Follicle miniaturization → Hair loss-AGA
	EDA2R	AR/EDA2R locus interaction [44]	In LD with AR locus; may regulate AR expression or ectodermal development pathways → Altered follicular development → ↑Miniaturization → Hair loss-AGA
	APCDD1	Wnt/β-catenin signalling Inhibition [46,53]	↑APCDD1 inhibits Wnt by binding LRP5/6 or Wnt ligands → ↓β-catenin activity → ↓Hair follicle regeneration → Miniaturization → Hair loss-AGA
	BMP2	BMP/TGF-β signalling [49,50]	BMP2 regulates follicle development; ↑BMP2 or SNPs → Telogen prolongation + Anagen inhibition → ↓Stem cell proliferation → Miniaturization → Hair loss-AGA
	BDNF (Brain-Derived Neurotrophic Factor)	Neurotrophin signalling [49,50]	↓BDNF expression or Val66Met variant → Disrupted follicle innervation and stem cell activity → Impaired anagen entry → Miniaturization → Hair loss-AGA
	EGR1	Transcriptional regulation [48]	↓EGR1 in frontal scalp → ↓Growth-promoting gene expression → ↓Follicle renewal → ↑Miniaturization → Hair loss-AGA
AA	STAT3, IL13, IL2RA, *etc.*	JAK-STAT autoimmune Inflammation [34]	↓Immune privilege → ↑IFN-γ/IL-15 → JAK1/JAK2 activation → STAT1/3 → ↑MHC on follicles → CD8^+^ T cell attack → Follicle destruction → Hair loss-AA

### Hair follicle stem cell dysfunction

Yuanhong Liu *et al.* [65] examined the dysregulation of hair follicle stem cells as a key mechanism in hair loss pathogenesis. Changes in the microenvironment, such as aging, diet, and stress, can disrupt the hair follicle stem cell lifecycle. The study identified mechanisms, including decreased lactate levels, mTORC2 deletions, and increased androgen receptor expression, as contributing factors. They suggested that stimulating hair follicle stem cells through various therapeutic interventions could potentially improve hair regrowth.

### Telomere length and genetic risk factors

Telomere length (LTL) has been identified as a potential genetic risk factor for AA. Yicheng Li *et al.* [66] conducted a Mendelian randomization study using GWAS data from the FinGen biobank and found that shorter leukocyte telomere length increased the risk of AA by 3.19 times. Their findings suggest that LTL could serve as a valuable genetic marker for predicting AA susceptibility and may help identify individuals at risk for early intervention.

### Single-nucleotide polymorphisms in androgenetic alopecia

It was reported in many studies that single-nucleotide polymorphisms (SNPs) are a promising contributor to AGA. SNPs directly affect gene expression and protein function, leading to changes in follicular development and alterations in androgenic pathways [32]. Hence, SNPs play a crucial role in understanding individual susceptibility to AGA and can also serve as promising targets for personalized medicine. SNPs in the PITX2 gene that cause AGA are the most distinctive feature identified in the Indian population. The most reported SNPs of the PITX2 gene are rs2200733, rs13143308, and rs10033464 and the associated molecular pathways, which are getting altered, are Wnt/β-catenin signalling pathway, androgenic receptor (AR) expression and 5α-reductase pathway [33]. Individuals with shorter repeats of SNP rs6152 exhibit higher AR activity, leading to AGA. The SNPs of the WNT10A gene -rs7349332 and SRD5A2 (rs523349) have direct impacts on the 5α-reductase functionality leading to hair follicle miniaturization [35]. Few researchers have also reported that SNPs modulate the prostaglandin pathway, thereby inhibiting or stimulating hair growth. PTGDR2 variants (rs533116, rs545659) and PTGFR-rs10782665 are among the variants that modulate the prostaglandin pathways. In addition to the above applications, some SNPs, such as PTGES2 (rs13283456) and ACE (rs4343), are used to analyse treatment responses, including minoxidil [35]. Additional genes involved in AGA are shown in Table 3.

**Table 3.** AGA-associated genes and SNP alterations

Gene	SNPs or Variants	Alterations
PITX2 [33]	rs2200733, rs13143308, rs10033464	Wnt/β-catenin signalling pathway, AR expression, and 5α-reductase
AR [34]	rs6152	Alters AR sensitivity
WNT10A [51]	rs7349332	Wnt signalling pathway
SRD5A2 [35]	rs523349	5α-reductase activity and DHT synthesis
PTGDR2 [32]	rs533116, rs545659	Inhibits the expression of PGD2 expression- prevents hair growth
PTGFR [37]	rs10782665	Enhances PGF2α activity-stimulates hair growth
PTGES2 [32]	rs13283456	minoxidil efficacy modulation-prostaglandin pathway alterations
ACE [32]	rs4343	Impacts the vascular response; minoxidil efficacy modulation
EBF1 [51]	rs929626, rs1081073	Immunity modulation

### Role of microRNAs in androgenetic alopecia

MicroRNAs (miRNAs) are key players in regulating the post-transcriptional gene expression [36]. Evidence from numerous studies indicates that miRNAs are involved in the regulation of hair loss [37]. The most commonly associated hair disorder associated with abnormal miRNA expression is AGA. Some of the pathways regulated by miRNAs include the Wnt/β-catenin signalling pathway, which promotes hair growth by inhibiting the DKK1 [5]. Increased levels of miR-133b prevent follicular growth. Besides individual microRNAs, a network comprising LncRNA-miRNA-mRNA has a significant role in the pathogenesis of AGA. 39 lncRNA AC010789.1 delays AGA progression by upregulating Wnt/β-catenin signalling and inhibiting miR-21-5p expression [67]. The researchers reported that miR-324-3p is absent in bald stem cells, making it a prominent marker for identifying the cause of hair loss [68]. Hence, it is clear that miRNA can be used in both the diagnosis and treatment of AGA. The role of miRNA in various pathways (both upregulation and downregulation) is tabulated below (Table 4).

**Table 4.** Role of microRNAs in AGA

miRNA	Expression in AGA	Primary Target(s)	Effect on Pathways	Functional Impact
miR-133b [69]	Upregulated	β-catenin	↓ Wnt/β-catenin signalling	AGA progression-hair loss
miR-29a/b1 [38]	Overexpression	Wnt and BMP pathway genes	↓ Wnt signalling	
miR-107 [38]	Downregulated	DKK1	↑ Wnt/β-catenin signalling	AGA suppression-hair growth
miR-21-5p [67]	Downregulated	Wnt pathway components	↑ HFSC proliferation via Wnt suppression	

### Polygenic risk scoring and Genome-wide Association Studies limitations

**Figure 5.**
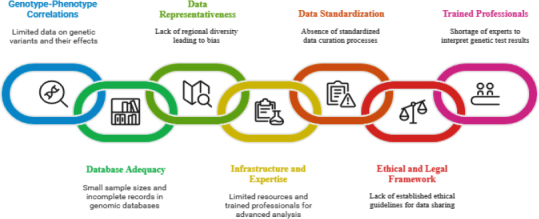
Limitations of GWAS scores in AGA prediction

Polygenic risk scores (PRS), which are part of Genome-wide Association Studies (GWAS), help analyse an individual’s genetic makeup and disease susceptibility by evaluating single-nucleotide polymorphisms (SNPs) [70]. PRS scores in AGA are used to determine individual susceptibility to hair loss and to analyse associated comorbidities, such as COVID-19. The severity of COVID-19 is directly associated with hair loss in individuals. Researchers have used pathway-based PRS (pPRS), which has helped to understand the correlation between AGA and COVID-19 [71]. Alterations in vitamin metabolism, WNT signalling, and aryl hydrocarbon receptor signalling have been observed. Some of the most common limitations observed in polygenic risk scoring are shown in Figure 5.

## Diagnostic biomarkers and technological advancements

Technological advancements in molecular diagnostics have led to the identification of new biomarkers for AA. Jiachao Xiong *et al.* [72] used gene expression and regulatory network analysis to identify four key genes, LGR5, SHISA2, HOXC13, and S100A3, as diagnostic biomarkers for AA. They recommended gene-targeted therapies such as ALECEPT and SIPLIZUMAB for patients with severe AA. In a similar vein, Qingde Zhou *et al.* [73] incorporated machine-learning techniques to identify immune biomarkers associated with AA. Their research identified SKAP1, PIK3CG, and CD8A as novel immune markers, which could be used for early diagnosis and targeted treatment of AA.

The literature indicates that AA is a complex, non-scarring form of hair loss and that multiple genes and factors are involved. Immune dysregulation, psychological stress, oxidative stress, and alterations in cytokine and chemokine signalling pathways also contribute to AA [74]. Hence, a combination of different clinical interventions will help treat AA.

Unlike AA, AGA is caused by androgens, such as dihydrotestosterone (DHT), resulting in hair loss and thinning specifically in the scalp's temporal and vertex regions [75]. AGA is an X-linked inherited disease, and symptoms typically develop over time. In this case, specifically, the anagen phase of the hair cycle is shortened, leading to hair thinning. Some of the genes involved in the androgen signalling pathway include the AR, FGF5, and EDA genes [75]. This gene may be a potential target for the treatment of AGA. In recent years, many researchers have focused on genetic analysis to predict the future risks of AGA. Few researchers have provided evidence by conducting Mendelian randomization studies to understand the relationship between metabolites and their impact on the risk of AGA. They concluded that α-tocopherol levels decrease and that supplementation to balance this reduces the risk of AGA.

## Tailored therapies for treating non-scarring alopecia

**Figure 6.**
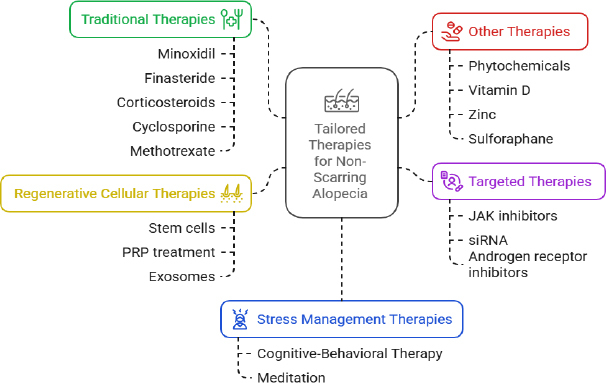
Tailored therapies for non-scarring alopecia

Various therapeutic strategies are being used by clinicians worldwide, from traditional to advanced regenerative treatments, to improve hair regrowth and prevent hair loss. Some of the treatments that are currently in use are shown below (Figure 6).

### Traditional therapies

Minoxidil is among the most commonly prescribed drugs for the treatment of AHA and promotes blood flow to hair follicles [76]. Minoxidil opens the potassium channels and extends the anagen phase. Finasteride is an androgen inhibitor that helps reduce hair thinning in AGA patients [77]. Both systemic and topical steroids to treat AA are currently in use; they are prescribed based on the severity of AA. Corticosteroids prevent the autoimmune effects on the hair follicles [74]. Restoration of the immune system can be done by administration of medications such as diphencyprone for patients who are suffering from severe symptoms of AA. Cyclosporine and Methotrexate are the medications that are administered last if the above medications are not working for patients suffering from severe symptoms of AA [78]. Even though these medications are effective in treating alopecia, there is a need to address the symptoms based on the individual's needs by using advanced technologies.

### Targeted therapies

Targeted therapies primarily target specific pathways that hinder hair growth. The JAK-STAT pathway is a signalling pathway involved in immune cell activation and in hair follicle destruction [79]. Tofacitinib and Ruxolitinib are the most commonly recommended JAK inhibitors that block JAK1 and JAK3, respectively, resulting in hair regrowth [80]. Other molecules are still in clinical trials, and targeting various molecular pathways with small-molecule inhibitors or enhancers will promote hair regrowth. Non-coding RNAs are involved in gene expression, and siRNA can be used to target AGA. This will help downregulate AR expression and prevent hair follicle miniaturization.

**Figure 7.**
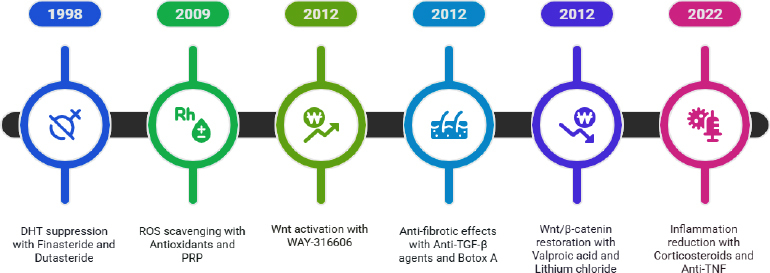
Advancements in AGA treatment strategies

In the past, symptom-based treatments were used to address the biological mechanisms of AGA-associated hair loss. The most significant breakthrough in the treatment of AGA was observed in 1998, when the administration of finasteride and dutasteride reduced DHT levels by inhibiting the enzyme 5α-reductase [81]. Oxidative stress is another factor contributing to AGA; hence, antioxidant treatments and PRP were recommended to neutralize free radicals and prevent the degeneration of follicular stem cells [82]. Hawkshaw *et al.* [83] reported that molecules such as WAY-316606 enhance expression of the Wnt/β-catenin signalling pathway, a pathway required for active hair growth. Similar studies were conducted by Bellani *et al.* [61], who summarised the importance of the Wnt/β-catenin, Sonic Hedgehog (Shh), BMP, and Notch pathways as promising targets for hair restoration. The usage of anti-TGF-β agents and even Botulinum toxin A to prevent follicular fibrosis has also gained importance. β-catenin stability can be modulated by valproic acid and lithium chloride, thereby promoting follicular stem cell regeneration. In recent years, corticosteroids and anti-TNF agents have been prescribed to minimize local immune inflammation, as inflammation in some cases can lead to AGA. Technological advances have shifted traditional therapies toward personalized approaches, treating diseases at the genetic or cellular levels rather than at the surface. The AGA treatment strategies from the past to the present are shown in Figure 7.

Many gene therapies are under clinical study, in which siRNA targeting regulates hair follicle development, and associated pathways include Wnt/β-catenin signalling and DHT receptors [38]. However, there exists a limitation of biological barriers.

### Regenerative cellular therapies

These are some of the most recommended alternative therapies to the traditional treatments of alopecia. Their mechanism of action is to stimulate the hair follicles and promote hair growth. Stem cell therapies: stem cells derived from adipose tissue, bone marrow, or the umbilical cord can be administered via intralesional injection for hair regrowth [2]. Stem cells modulate growth factors such as VEGF and IGFBP-1, which enhance blood flow to hair follicles and restore hair growth [84]. PRP is the second most widely used regenerative hair follicular therapy, in which the patient's plasma, rich in platelets, growth factors, and cytokines, is injected into the scalp to promote hair growth [84]. Exosome therapy is a cell-based regenerative therapy that promotes follicular regeneration [85].

**Figure 8.**
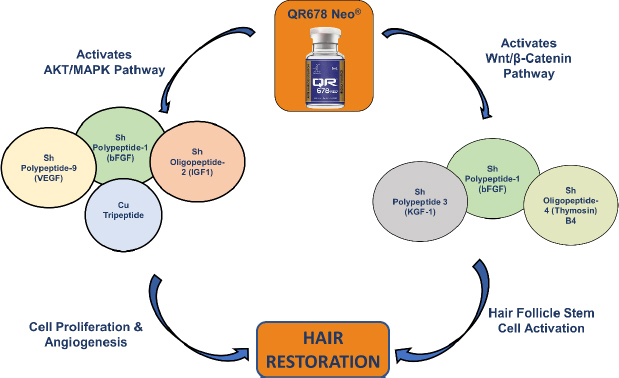
Role of QR678 Neo® in non-scarring alopecia [90]

Novel biomimetic peptides and growth factor-based formulations such as QR678® have demonstrated efficacy in promoting hair follicle regeneration and represent a significant advancement in non-invasive treatment approaches [61,78-92]. Preclinical and clinical investigations have consistently demonstrated the safety and efficacy of QR678 Neo® in diverse alopecia phenotypes [91-101]. As shown in Figure 8, QR678 Neo® demonstrates therapeutic efficacy by targeting the Wnt/β-catenin and AKT/MAPK pathways, which are essential for activating follicular stem cells, promoting angiogenesis, and prolonging the anagen phase. This highlights the role of QR678® as a comprehensive, pathway-based regenerative therapy for hair restoration. Recent clinical investigations indicate that microneedling and dermaroller-assisted delivery significantly enhance scalp penetration and efficacy of QR678 Neo®, with trials confirming superior follicular regeneration compared with minoxidil and finasteride, especially as monotherapy [102-105]. In addition to its role in alopecia management, QR678 Neo® has also been validated as an intraoperative adjunct in follicular unit extraction, where its use as a graft-holding medium significantly improved follicle viability and donor site recovery [106].

Integration of artificial intelligence and machine learning models holds further potential for improving early diagnosis and treatment planning. Even though some of these methods are currently in use, there is no standardization of isolation, injection dosage routes, etc., which limits their efficacy.

### Tailored therapies based on single nucleotide polymorphisms

SNP identification helps to tailor treatment for AGA based on the individual's genetic makeup, thereby minimizing side effects. Individuals who have SRD5A2 polymorphisms exhibit varied responses to finasteride and dutasteride [35]. The same can be observed in people who have SNPs in the PTGES2 and ACE genes for other drugs, such as minoxidil. Few studies have reported that individuals with favourable variants show significant regrowth, whereas those without these variants may not support hair growth. It was also reported that prostaglandin-based therapies, which are also based on SNPs, are in use for hair treatment. Individuals with higher levels of PGD2 expression favour hair regrowth when treated with PGD2 inhibitors [85]. It is recommended that for patients suffering from existing comorbidities, there is a need to study both metabolic profiling and genetic makeup (SNPs) to bring about positive outcomes.

### Other therapies and treatments

Phytochemicals such as decursin, ginsenosides, rosmarinic acid, sulforaphane, etc., are being used for hair regeneration [87]. Various studies also reported that certain psychological comorbidities, such as stress, significantly impact hair growth; hence, cognitive-behavioural therapy (CBT) and meditation are recommended to reduce stress and prevent the recurrence of AA [88]. Advances in gut microbiome analysis and associated supplements have also been shown to improve hair growth [83]. In addition, integrating technological advancements into diagnostic methods will enhance treatment.

## Concluding remarks and future directions

Non-scarring alopecia, encompassing conditions such as androgenetic alopecia (AGA) and alopecia areata (AA), is a complex, multifactorial disorder influenced by genetic predisposition, immune dysregulation, and environmental stressors. A comprehensive understanding of individual genetic makeup and molecular alterations - particularly within key pathways such as JAK-STAT and androgen receptor signalling can facilitate the development of personalized therapeutic strategies for effective hair regrowth.

Advancements in genomic technologies, including genome-wide association studies (GWAS) and next-generation sequencing (NGS), are increasingly employed to identify genetic markers predictive of early-onset alopecia. Moreover, elucidation of the intricate interplay among immune dysfunction, oxidative and psychological stress, hormonal imbalances, and comorbid conditions provides deeper insight into disease pathophysiology.

Emerging therapeutic modalities, including platelet-rich plasma (PRP), stem cell therapy, exosome-based treatments, corticosteroids, and gene-editing technologies such as CRISPR, show promising results. Integration of artificial intelligence and machine learning models holds further potential for improving early diagnosis and treatment planning.

However, current therapeutic approaches face challenges related to standardization, delivery methods, and biological barriers. Future research should focus on overcoming these limitations through the optimization of combination therapies and multimodal approaches to enhance clinical outcomes and ensure long-term remission in patients with non-scarring alopecia.

By 2050, due to rapid advances in technology integration, trichology may revolutionize treatments through precision medicine, in which therapies will be tailored to each individual’s genetic profile. Patients diagnosed with AR/SRD5A2 variants will receive treatments targeting androgen receptors, while those with mutations in the Wnt pathway may benefit from DKK-1 inhibitors or Wnt activators. Similarly, individuals diagnosed with AGA due to other pathways, metabolic factors, or microbiome alterations will receive tailored targeted therapies.

Technologies such as NGS, polygenic scoring, and AI-based tools will help clinicians create digital twins of patients, enabling simulation of treatments before actual therapy. The future of trichology will focus on translating genetics at the molecular level into individualized therapies, ensuring lifelong follicular resilience and reshaping the field of hair care treatment.
